# Determining the effects of maternal upright birth position on maternal and foetal outcome among pregnant women delivering in government hospitals in Dar es Salaam: A randomized controlled trial study protocol

**DOI:** 10.1097/MD9.0000000000000285

**Published:** 2023-07-25

**Authors:** Sospeter Mkangi, Saada A. Seif, Rehema Bakari, Aisha Tambwe

**Affiliations:** a Department of Clinical Nursing, The University of Dodoma, Dodoma, Tanzania; b Department of Nursing Management and Education, The University of Dodoma, Dodoma, Tanzania.

**Keywords:** birth position, delivery, pregnant women, randomized control trial, Tanzania, upright

## Abstract

A prolonged active stage of labour leads to complications and adverse effects for both mother and newborn, and among of it causes is a horizontal birth position, which accounts for 91.4% to 99.7% of all vaginal births in Tanzania. The study purpose is to determine the effect of the upright birth position on reducing the duration of the second stage of labour, perineal tear, maternal birth satisfaction, and apgar score among women delivering in government hospitals in Dar es Salaam-Tanzania.

**Methods:**

This will be a randomized controlled trial that will include 116 participants in experimental and 116 participants in the control group. A simple random sampling will be used to select 4 out 6 hospitals with adjustable beds in Dar es Salaam. In each hospital, participants will be randomly allocated to an either experimental group or control group whereby, the experimental group will be directed and helped to assume an upright birth position during the second stage of labour while the control group will be allowed to assume the horizontal position. Data will be analysed using SPSS version 28. An independent *t* test and one-way MANOVA will be used to compare the effects of intervention between the experimental and control, and a *P* value of < .05 will be regarded as a statistically significant difference.

**Discussion:**

The study is important in improving the birth outcome while putting a high consideration to the mothers’ birth satisfaction.

## 1. Introduction

Maternal morbidity and mortality remain a big global health challenge. The report shows in 2017, 295,000 women died from preventable maternal deaths and 94% of these deaths occurred in low resources countries whereby Sub Saharan African countries contributed 66%,.^[1]^ In Tanzania, maternal mortality is 524 per 100,000 live births.^[2,3]^ Most maternal morbidity and mortality are caused by postpartum haemorrhage, sepsis, unsafe abortion, pre-eclampsia, eclampsia, and obstructed labour.^[4–6]^ Obstructed labour is said to result in prolonged labour, which occurs when the active phase of labour is longer than 12 hours. About 8.5% to 21.7% of women giving birth are affected by a prolonged active phase of labour, and the incidence is 2 to 3 times higher among primipara compared to multipara.^[7–9]^ The prevalence of prolonged labour in Tanzania is 27.1%.^[10]^

The prolonged active stage of labour can lead to complications and adverse effects for both mother and newborn, it is associated with postpartum haemorrhage, ruptured uterus, puerperal sepsis, urine incontinence, episiotomy, and perineal tear, assisted vaginal delivery, and caesarean section. In newborn, it can cause birth injuries, low apgar score, and admission to the intensive care unit.^[11–13]^ Reports in Nigeria showed that prolonged labour leads to 80.97% caesarean section, 14% laparotomy, 4.9 % destructive operations, 16.5% sepsis, 13% ruptured uterus, 7.81% puerperal sepsis, 3.9% postpartum haemorrhage, 0.98% maternal mortality, 28.8%, low apgar score (<7), and 35.15% perinatal mortality.^[14]^ In Ethiopia, 38.05% sepsis, 38.08% stillbirth, 35.4% postpartum haemorrhage, 29.8% ruptured uterus 29.8%, and maternal 17.27% mortality.^[15]^ Furthermore, in Tanzania prolonged labour causes 94% caesarean section 3.5% vacuum delivery, 9 % postpartum haemorrhage, 16% low apgar score, and 8.8% stillbirth.^[16]^

The prolonged second stage labour is caused by problems with, the passenger, birth canal, uterine contraction, psychological state of the mother, and maternal birth position.^[17]^ The passenger factors like malposition, congenital anomalies, and big size compared to maternal pelvic size (cephalopelvic disproportion),^[17,18]^ and the inadequate uterine contractions that cannot produce power enough to push the baby through the birth canal^[19]^ causes the prolongation of the second stage of labour. The horizontal birth position is also linked to a prolonged second stage of labour as it limits pelvic outlet diameter by restricting coccyx movement, sacrum flexibility, and widening of the pubis symphysis.^[20–25]^ Even though the horizontal birth position is among the causes of prolonged labour, it is the most practiced birth position in Tanzania, and it counts for 91.4% to 99.7% of all vaginal births.^[22,26]^

To prevent incidences of prolonged labour and its associated complications, Tanzania has done several efforts including increasing birth coverage by skilled birth attendants by employing and training medical staff, ensuring the availability of adequate supplies and equipment required during labour and delivery, introducing free of charge labour and delivery services, formulation of guidelines for obstetric care, as well as introducing of maternal and perinatal death surveillance response program.^[27–29]^ Furthermore, she has adopted world health organization recommendations such as augmentation of labour, and the use of patograph in monitoring labour. Despite these efforts, still, the incidences of prolonged labour have remained quite higher, therefore the purpose of this study is to determine the effect of the upright birth position on reducing the duration of the second stage of labour, perineal tear, maternal birth satisfaction, and apgar score of neonates among women delivering in government hospitals in Dar es Salaam.

## 2. Objectives

This study is aimed to determine the effectiveness of the upright birth position on reducing the duration of the second stage of labor, perineal tear, maternal birth satisfaction, and apgar score of neonates among women delivering in government hospitals in Dar es Salaam, Tanzania.

## 3. Methods: participants, interventions and outcomes

### 3.1. Study setting

This study will be conducted in the selected government hospitals, of secondary and tertiary levels in Dar es Salaam. According to the ministry of health in 2022, Dar es Salaam has a total number of 1199 of operating healthcare facilities, among them, 210 are owned by the government, of which there are 155 dispensaries, 32 health centres, 18 hospitals, and 5 laboratories and clinics (The united republic of Tanzania ministry of health, 2022). This study will be conducted in hospitals with adjustable beds, among the 18 hospitals, only 6 have the adjustable beds.

### 3.2. Study design

**Figure 1.**
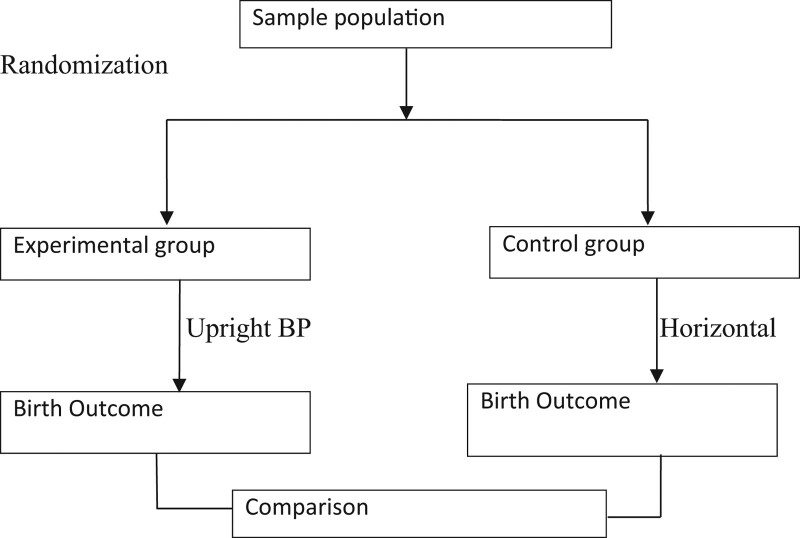
Diagram of study design. BP = birth position.

A randomized controlled trial will be used. There will be 2 groups in this study, the experimental and the control group. The participants will be randomly allocated to an either experimental group or control group. The experimental group will be directed and helped to assume an upright birth position during the second stage of labour while the control group will be in the horizontal position. The birth outcomes which are the duration of the second stage of labour, perineal tear, maternal birth satisfaction, and apgar score will be compared between the experimental group and the control group as shown in “Figure 1.”

The study population will be all pregnant women who will be delivering in the government hospitals where this study will be carried out. To be included as a participant in this study, the following criteria should be met: term pregnancy, age between 20 and 35 years, singleton pregnancy, cephalic presentation, vaginal delivery is not contraindicated, spontaneous labour, nulliparous, multiparous, no history of any previous uterine surgery, mentally fit, in the latent phase of the first stage of labour, no surgical indication at the time of study beginning, stature ≥150 cm. The woman will be considered ineligible to participate in this study if she has either of the following characteristics: pregnancy complicated with anaemia, pregnancy-induced hypertension, diabetes, and foetal congenital malformations, grand parity (para 5 or more), history of previous assisted vaginal delivery, have more than 5 years from the last delivery, and who will refuse to participate.

### 3.3. Sample size estimation

The minimum sample size for this study will be determined using the following formula as suggested by previous studies when researchers conduct on the sample size estimation for the clinical trials.^[30]^ Proportion to reach the desired minimum sample size has been adopted from a previous scholar,^[31]^ who did a study on the effect of flexible sacrum position on maternal and neonatal outcomes in public health facilities in Ethiopia who found that the proportion of the short duration of labour was 84.4% and 95.9% in control and experimental groups respectively.


$$\text{n}=2{\left(\text{z}\alpha +\text{z}1-\beta \right)}^{2}\cdot PQ/d^{2}$$

whereas n = the Minimum sample size of the study, P_1_ = the proportion of shortened duration of the second stage of labour in horizontal position 84.4 adopted from ^31^, P_2_ = The proportion of shortened duration of the second stage of labour in upright birth position 95.9 ^31^, *P* = Effect size (P_2_ + P1)/2 = (95.9 + 84.4.)/2 = 90.15, *Q* = 100 – *P* = 100–90.15 = 9.85%, *d* = effect size, P_2_–P_1_ = 95.9–84.4 = 11.5%, ${}^{Z_{1-\alpha }}{/}_{2}=1.96$, the value of the standard normal distribution corresponding to at confidence level of 95% and significance level of 5% (2 sided), ${}^{Z_{1-\beta }}{/}_{2}=0.84$, the value of standard normal distribution to desired level of power of (80%). The calculated sample size is 105 for each group. Adding the 10% of attrition rate, the total sample size for this study is 232, with 116 participants in each group.

### 3.4. Sampling technique

A proportionate sampling will be used to allocate number of the study participants for each healthcare facility depending on the number of deliveries per month. In each healthcare facility, a randomization will be conducted to assign participants to either experimental or control group in the ratio of 1:1.

### 3.5. Intervention description

#### 3.5.1. For the experimental group.

The intervention will be divided into 3 phases;

##### 3.5.1.1. Phase I (orientation phase).

In this phase the study participants will be oriented to different upright birth positions. While still in the latent phase of the first stage of labour, a researcher or research assistant will teach the participants about the upright birth positions. The client centered approach will be used whereby every participant will be taught individually about the upright birth position based on meaning, types, advantages, and how to assume a different upright position. This phase will be divided into 3 parts which are, the introduction, main body, and revision.

The first part will be an introductory part which will take 3 minutes; in this part, a researcher will ask the participant what she knows about the upright birth position. The researcher will have a paper where the question is written. The second part is the main body, this section will take 15 minutes. In this part with aid of printed pictures of a woman delivering different upright birth positions. The researcher will teach respondents the advantages and assist them in how to assume the positions.

In the squatting position, while standing on the delivery bed with both legs apart and knees flexed, the client will hold metals on the left and right sides of the delivery bed and the trunk will be kept straight upward however she may lean forward or backward to increase flexibility.

For the kneeling position participant will be told to kneel on the delivery bed then she will lean forward and supports herself on the palms of her hands. Also, the client will be taught that when she will feel the urge to push, she will do so while keeping the upright birth position under the assistance of the researcher, however she can assume any other position between contractions. In hands knees position, each participant will be taught and assisted to assume the on all 4 positions during the second stage, when there is no contraction participant will be allowed to rest in any position and the process will be repeated until the baby delivered.

Third part, this will be is revision part, it will take 3 minutes. In this part the respondent will be asked to tell the importance of the upright birth position and practice by assuming either of the upright birth position that she will feel to elaborate. This will be the last part of orientation phase, then mother will wait until the second stage of labour reaches. The second stage of labour will be defined as the period from the time of full cervical dilatation to delivery of baby.

##### 3.5.1.2. Phase II.

As the participant reach the second stage of labour, the researcher will record the time and then will encourages and assist mother to assume either any upright birth position when she feels urge to push during contraction until delivery of the baby. Between contractions respondent be allowed to rest in any comfortable position, however during pushing she will return to upright position which she chosen.

Throughout the second stage, maternal vital signs, labour progress, and fetal heart rate will be monitored. Fetal heart rate, uterine contraction and maternal vitals will be monitored at interval of fifteen minutes, thirty minutes and an hour respectively. In case during the study any condition that will need emergence intervention such as fetal distress, obstructed labour, prolonged labour, and cord prolapse, arise the condition will be managed according to the hospital protocol. Augmentation by oxytocin, assisted vaginal delivery by low vacuum extraction and episiotomy will be performed only if a necessary indication to do it. For either of the circumstance above, the participant will be excluded from the study however she will be reported.

##### 3.5.1.3. Phase III.

This will be the assessment part. Immediately after the delivery, the delivery time will be recorded, the newborn will be scored according to apgar score in the first and fifth minutes. Then the active management of third stage of labour will be done according to hospital protocol. The perineum will be inspected to rule out perineal tear, in case there will be perineal tear, it will be classified according to ACOG then repaired, the amount of blood loss will be quantified for estimation. Finally, the outcomes will be recorded. For the newborn, the outcomes will include apgar score in first and fifth minutes, admission in intensive care unit, and early neonatal death. The maternal outcome will include the duration of the second stage of labour, perineal tear, and maternal satisfaction.

#### 3.5.2. For the control groups.

The researcher will monitor the progress of labour while client is in the horizontal position, as the client reaches the second stage of labour, time will be recorded and mother will be assisted to deliver in the horizontal position. Fetal heart rate, labour progress, and maternal conditions will be monitored. And in case, any condition that need emergence intervention arise it will be managed as per hospital protocol. For those who will deliver successfully vaginally, upon completion of the second stage of labour, the newborn will be scored according to the apgar score in the first and fifth minutes post-delivery. Then the third stage of labour will be managed, the perineum will be assessed to rule out a perineal tear and in case there will be a sustained perineal tear, it will be classified according to ACCOG. Outcomes under the study will be recorded. For the newborn, the outcomes will include apgar score in first and fifth minutes, and for maternal, the outcome will include the duration of the second stage of labour, perineal tear, and maternal satisfaction

### 3.6. Measurement of variables

#### 3.6.1. Dependent variables.

The dependent variables in this study will includes;

*Maternal outcome*: In this study maternal outcomes will include, duration of the second stage of labour, perineal tear, and maternal satisfaction.

*The duration of the second stage* of labour will be measured in minutes by using a patograph. Duration of the second stage of labour will be categorized into 4 groups, <15 minutes, 15 to 30 minutes, 31 to 60 minutes, 61 to 90 minutes, 91 to 120 minutes, 121 to 180 minutes, and ≥ 181 minutes. In prime gravida the mean duration of the second stage of labour within 3 hours will be considered normal, however, any second stage of labour that will extend beyond 3 hours will be prolonged second stage of labour. Conversely, in multipara participants the mean duration of the second stage of labour within 2 hours will be normal while beyond 2 hours will be prolonged second stage.

*Perineal tear* will be measured by the presence of laceration in the perineum, vagina, anal mucosal and sphincters. The absence of a tear will be termed as intact perineum, in case there will be a tear, it will be classified into degrees according to the world health organization standardized obstetric diagnostic code (ICD-10 Version:2019). First degree tear is the laceration of the superficial vaginal mucosa with or without the perineal skin. In the second degree, the laceration involves the vaginal mucosa and the perineal body. The third degree tear, the laceration involves the vaginal mucosa, perineal skin and the anal sphincter. In fourth degree perineal tear the laceration involves third degree tear plus rectal mucosa.

*Satisfaction*. The satisfaction will be measured by 2 items using a Likert scale. participant in each group will be asked 2 questions each question will have 5 possible options; the first question will ask the patient level of satisfaction on birth position she used during this study and second question will ask about the birth position of participant would choose to assume during child birth in consecutive pregnancies. The options will be scored from 1 to 5, the highest score will be 5 and will imply highest satisfaction, (5 = very satisfied, 4 = satisfied, 3 = neither dissatisfied nor satisfied, 2 = dissatisfied and 1 = very dissatisfied.

*Newborn outcome*. The newborn outcome will be measured by considering newborn apgar score. The Apgar score will be measured in the first and fifth minutes following the delivery of the baby, basing summation of the score 5 parameters; heart rate, respiration rate, muscle tone, body colour, and reflex activity. Then the apgar score will be categorized according to ACOG and AAP whereas a score between 7 to 10 will be considered reassuring, 4 to 6 is moderately abnormal and 0 to 3 is low.

#### 3.6.2 Independent variables

Sociodemographic characteristics: This will be measured by using 4 items (marital status, education level, and occupation and age).

Obstetric characteristics: This variable will be measured by using 9 items (gravidity, parity, gestation age and antenatal visits, number of living children, history of abortion abortion, antenatal care visits, gestation age, the expected date of delivery history of chronic illness, pregnancy and birth related to the previous pregnancy, and current pregnancy medical problems.

Birth position: The birth position will be measured by recording the type of position that participant will assumes during the second stage of labor, whether upright or horizontal.

### 3.7. Data collection methods

Data will be collected by using 2 methods, interviewer-administered questionnaire and observation. The questionnaire will be used to collect information on sociodemographic and obstetric characteristics and satisfaction on the birth positions. The observation will be used to collect information on the duration of the second stage of labour, the state of the perineum and newborn apgar score. Throughout the study, the principal investigator will supervise research assistants to make sure that data collection is done as planned and consistently for every participant by ensuring that for every participants consents before being involved in the study, questions provides the same meaning among all respondents, questionnaires are filled correctly, no harm, privacy and confidentiality are maintained, every participant receive quality care intrapartum care throughout time she will remain under the study.

### 3.8. Data collection tools

The observation checklist will be adopted from Badi et al,^[31]^ and modified to fit the purpose of this study. The observation checklist will have fifteen items. These items will be used to assess birth position used during second stage of labour, the duration of second stage of labor, perineal tear, and apgar score. The structured questionnaire will be adopted from Berta et al.^[32]^

### 3.9. Data management plans

Hard copies documents will be kept in safe place, and electronic documents will be kept in email and drop box accounts to which only principal investigator have a password for access. Other soft copy materials will be kept in personal laptop protected with password.

### 3.10. Data analysis

The Data will be analyzed using SPSS version 28. The data will be cleaned and checked for completeness before being subjected to models of analysis. Descriptive statistics will be used to describe the background characteristics of participants. An independent *t* test and one-way MANOVA will be used to compare the effects of intervention between the experimental and control, and a *P* value of < .05 will be regarded as a statistically significant difference.

## 4. Ethical consideration

Ethical clearance was obtained from the Institutional Research Review Ethics Committee (IRREC) of the University of Dodoma. Then permission to conducting this study in hospitals will be obtained from regional medical officer and from the managing director. Before recruitment in the study the informed consent shall be obtained from the participants after being well informed about the purpose of the study, how it shall be conducted, anticipated impacts and all alternatives. During the study, participants will have freedom to withdraw from the study at any time. To ensure privacy, no information that reveals participant identity will be included instead all participants will be assigned numbers for anonymity. Furthermore, confidentiality will be ensured by keeping information obtained from the participant safely.

### 4.1. Status and timeline of the study

The study is expected to be conducted for a period of 3 months, and on the day of submission, the status is completion of the proposal development.

## 5. Discussion

### 5.1. Strength and limitation of the study design

The strength of this study is the use of a randomized controlled trial, which is the most rigorous design of determining the cause-effect relationship for the purpose of assessing cost effectiveness of the intervention. To the best of the authors’ knowledge, this will be the first study in Tanzania. Hence, the findings from this research will help to determine the feasibility of the intervention in our local setting. But also, will lay a foundation for the other researchers to do researches on the effects of the upright birth position on the other maternal and neonatal outcomes. The limitations of this study will be the availability of midwives who are competent in assisting a woman to deliver in upright position, as majority in our settings are not used to this position. To overcome this, there will be a 1 training to all midwives from the hospitals where this research will be conducted.

## Acknowledgments

We acknowledge the University of Dodoma for educational support which resulted to the production of this protocol.

## Author contributions

**Conceptualization:** Sospeter Mkangi.

**Methodology:** Sospeter Mkangi, Rehema Bakari, Saada A. Seif.

**Supervision:** Saada A. Seif.

**Writing – original draft:** Sospeter Mkangi, Saada A. Seif.

**Writing – review & editing:** Rehema Bakari, Aisha Tambwe.

## Correction

In section 3, Independent Variables was mislabelled as 3.7 and has been corrected to 3.6.1. Subsequent sections have been renumbered correctly.
